# Functional expression of a novel methanol-stable esterase from G*eobacillus subterraneus* DSM13552 for biocatalytic synthesis of cinnamyl acetate in a solvent-free system

**DOI:** 10.1186/s12896-020-00622-1

**Published:** 2020-06-29

**Authors:** Xianghai Cai, Lin Lin, Yaling Shen, Wei Wei, Dong-zhi Wei

**Affiliations:** 1 State Key Laboratory of Bioreactor Engineering, Newworld Institute of Biotechnology, East China University of Science and Technology, 130 Meilong Road, Shanghai, 200237 People’s Republic of China; 2 Shanghai University of Medicine and Health Sciences, Shanghai, 200093 People’s Republic of China; 3 Research Laboratory for Functional Nanomaterial, National Engineering Research Center for Nanotechnology, Shanghai, 200241 People’s Republic of China

**Keywords:** *Geobacillus subterraneus*, Esterase, Methanol-stable, Biocatalysis, Cinnamyl acetate

## Abstract

**Background:**

Esterases are widely distributed in nature and have important applications in medical, industrial and physiological. Recently, the increased demand for flavor esters has prompted the search of catalysts like lipases and esterases. Esterases from thermophiles also show thermal stability at elevated temperatures and have become enzymes of special interest in biotechnological applications. Although most of esterases catalyzed reactions are carried out in toxic and inflammable organic solvents, the solvent-free system owning many advantages such as low cost and easy downstream processing.

**Results:**

The gene *estGSU*753 from *Geobacillus subterraneus* DSM13552 was cloned, sequenced and overexpressed into *Escherichia coli* BL21 (DE3). The novel gene has an open reading frame of 753 bp and encodes 250-amino-acid esterase (EstGSU753). The sequence analysis showed that the protein contains a catalytic triad formed by Ser97, Asp196 and His226, and the Ser of the active site is located in the conserved motif Gly95-X-Ser97-X-Gly99 included in most esterases and lipases. The protein catalyzed the hydrolysis of *p*NP-esters of different acyl chain lengths, and the enzyme specific activity was 70 U/mg with the optimum substrate *p*NP-caprylate. The optimum pH and temperature of the recombinant enzyme were 8.0 and 60 °C respectively. The resulting EstGSU753 showed remarkable stability against methanol. After the incubation at 50% methanol for 9 days, EstGSU753 retained 50% of its original activity. Even incubation at 90% methanol for 35 h, EstGSU753 retained 50% of its original activity. Also, the preliminary study of the transesterification shows the potential value in synthesis of short-chain flavor esters in a solvent-free system, and more than 99% conversion was obtained in 6 h (substrate: cinnamyl alcohol, 1.0 M).

**Conclusions:**

This is the first report of esterase gene cloning from *Geobacillus subterraneus* with detailed enzymatic properties. This methanol-stable esterase showed potential value in industrial applications especially in the perfume industry.

## Background

Lipolytic enzymes are widely distributed and are abundantly found in animals, plants and microorganisms. True lipases catalyze the synthesis and hydrolysis of relatively long-chain acylglycerols, and in contrast, esterases catalyze glycerol esters with acyl chain lengths of < 10 carbon atoms [1]. Microbial esterases have broad substrate specificity, require no cofactor, exhibit high regio- and stereo specificity, and have important applications in physiological, industrial and medical [2]. Flavor esters have been widely used in food, pharmaceuticals, flavors and perfume. Traditionally, flavor esters can be synthesized by chemical method or obtained through natural sources extraction. In recent years, enzymatic synthesis has aroused widespread interest and has obvious advantages such as environmental friendliness, high efficiency and moderate reaction condition. Recently, the search of catalysts like lipases and esterases has been prompted by the increased demand of flavor esters.

Thermophiles are a valuable source of thermostable enzymes with properties that are often associated with stability in solvents and detergents, giving these enzymes considerable potential for many biotechnological and industrial applications. Thermostable esterases shows unique advantages in the detergent industry/organic synthesis and have become important enzymes of special interest in biotechnological applications [3]. So, the cloning of novel esterase genes (with distinct features such as thermostability or catalyst reaction) is also an important research field. Esterases from *Geobacillus* species have attracted more attention since they have thermostability and the potential used in food and chemistry industries. Montoro-García S et al. described the cloning and characterization of a thermostable carboxylesterase from *G. kaustophilus* HTA426 [4]. The cloning, purification and characterization of thermostable esterase and a lipase from *G. thermoleovorans* YN was described [5]. The gene form *G. thermoleovorans* CCR11 thermostable lipase was cloned with enzymatic properties [6]. A thermophilic lipase gene of *G. stearothermophilus* JC was cloned and expressed in a pET-28a expression vector [7]. The immobilized recombinant lipase from *G. stearothermophilus* G3 was used to produce fatty acid methyl esters [8]. A member of a new family of carboxylesterases from *G. thermodenitrificans* was cloned and characterized [9]. Although some *Geobacillus* sp. lipases genes from *G. kaustophilus*, *G. thermoleovorans*, *G. stearothermophilus*, *G. thermocatenulatus* and *G. thermodenitrificans* have been cloned and sequenced, there is no report of wild strain esterase activity and esterase gene cloning from *G. subterraneus*.

In this study, a novel esterase gene was obtained from *Geobacillus subterraneus* DSM13552 and expressed in *E. coli*, and the enzyme characterizations including activity/stability, optimum pH, optimum temperature and substrate specificity were also described. Meanwhile, the cinnamyl acetate production using whole-cell biocatalyst was studied with preliminary results in this research. This is the first report of esterase gene cloning from *G. subterraneus* with detailed enzymatic properties. This methanol-stable esterase showed potential value in industrial applications especially in the perfume industry.

## Results

### Cloning and sequence analysis of the gene

**Fig. 1:**
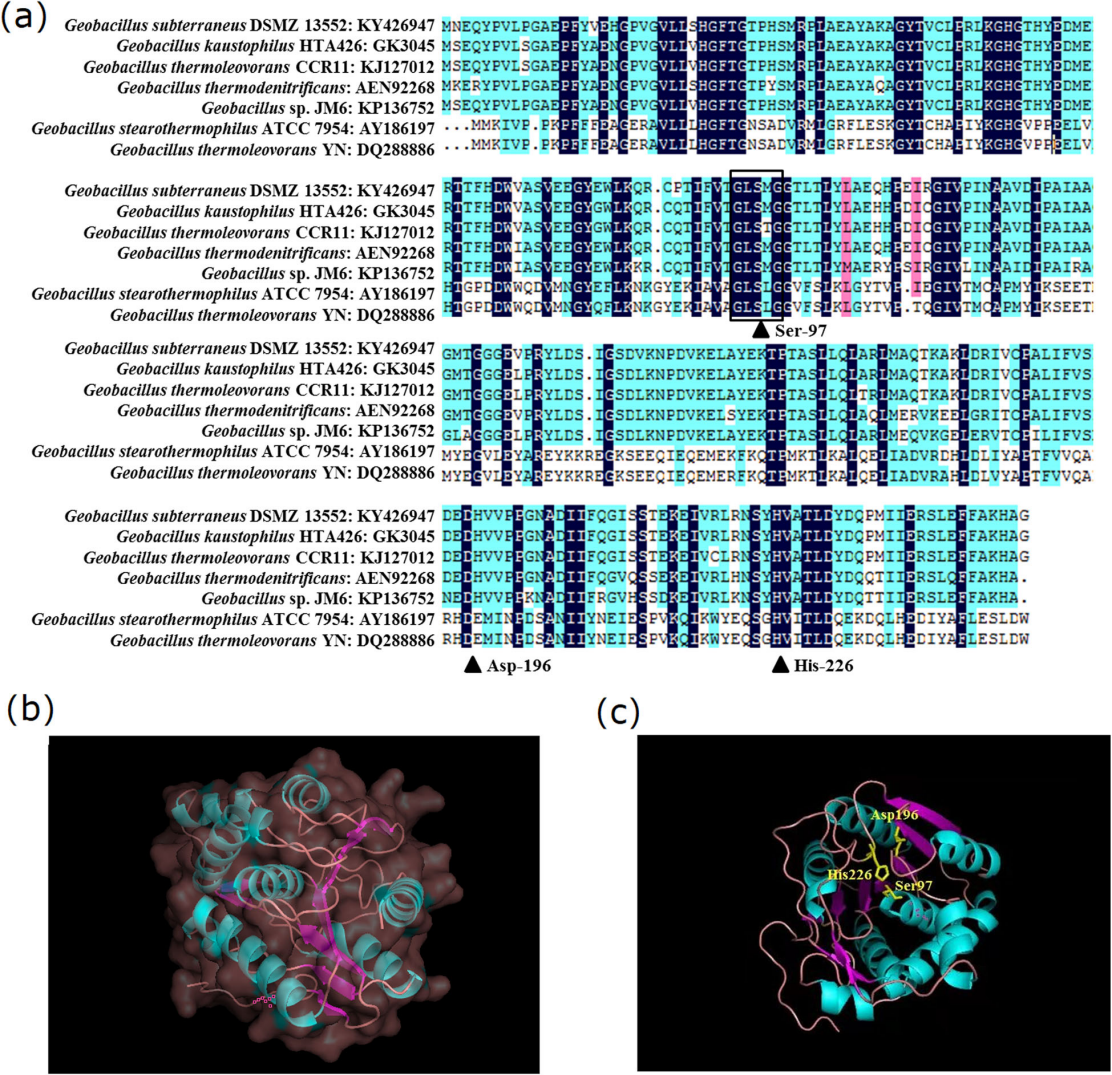
Conserved sequence alignment and structure analysis of EstGSU753. (a) Conserved sequence alignment of EstGSU753. The structures are denoted as follows: ▲, the catalytic site (Ser-97, Asp-196 and His-226). The conserved region, Gly-Xxx-Ser-Xxx-Gly was boxed. (b) The whole three-dimensional structure. The three-dimensional structure of Est_BAS_ was predicted by the SWISS-MODEL server. (c) The catalytic site of Est_BAS_ structure. The catalytic triad: Ser-97, Asp-196 and His-226 residues are marked in gray

The esterase gene *estGSU*753 was amplified by primers GSU753-EU/GSU753-ED. Sequence analysis revealed the presence of a 753 bp ORF encoding a polypeptide of 250 amino acid residues corresponding to a molecular weight of 27.5 kDa, and the pI value was calculated to be 5.44 by the ExPASy compute pI/Mw program algorithm. The *estGSU*753 ORF uses ATG as the start codon and the G + C content (%) is 48.3 mol %. Homology analysis revealed that EstGSU753 in *G. subterraneus* shared the most identity with carboxylesterase in *G. kaustophilus* HTA426 (GI: BAD77330) and 94% identical to the carboxylesterase in *G. thermoleovorans* CCR11 GI: KJ127012 (90% to *G. thermodenitrificans* GI: AEN92268, 86% to *Geobacillus* sp. JM6 GI: KP136752, 34% to *G. stearothermophilus* ATCC 7954 GI: AY186197 and 33% to *G. thermoleovorans* YN GI: DQ288886) (Fig. 1a). Also, EstGSU753 contains a single catalytic domain of the alpha/beta hydrolase family and belongs to the family of esterase (EC 3.1.1.1). The catalytic triad Ser-97, Asp-196 and His-226 residues were seen in the regions. The conserved region, Gly-Xxx-Ser-Xxx-Gly (the feature of the lipase sequences from *Bacillus* sp.) is boxed. As this esterase had not been previously reported, the three-dimensional structure of EstGSU753 was predicted by the SWISS-MODEL server and the protein structure of EstGSU753 was generated with a specific template (PDB code: 4kea, 94% identical) (**Fig. 1bc**), which was viewed by PDB Viewer.

### Expression and purification of the recombinant enzyme

**Fig. 2:**
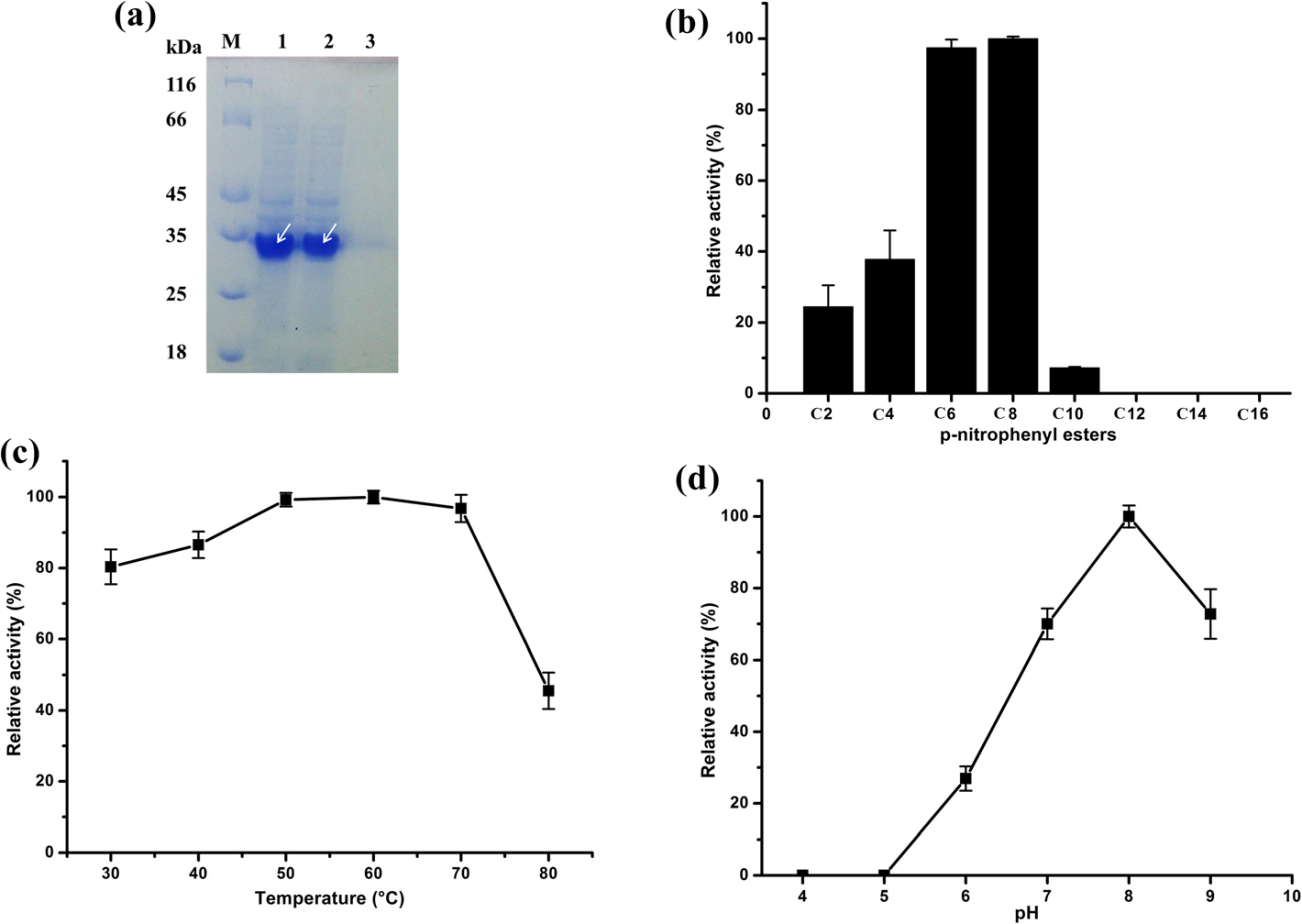
SDS-PAGE analysis and enzymatic properties of EstGSU753 expressed in *E. coli*. (a) SDS-PAGE analysis of EstGSU753 expressed in *E. coli*. (IPTG concentration: 0.1 mM, induction time: 20 h). Lane M: protein marker; lanes 1–3: cell lysate, supernatant and purification of EstGSU753. (b) Determination of the optimum substrate for recombinant Est_BAS_ΔSP. *p*NP-acetate (C2), *p*NP-butyrate (C4), *p*NP-hexanoate (C6), *p*NP-caprylate (C8), *p*NP-decanoate (C10), *p*NP-laurate (C12), *p*NP-myristate (C14) and *p*NP-palmitate (C16) (c) Effect of temperature on the activities (pH 8.0). (d) Effect of pH on the activities (60 °C)

Recombinant strain was grown in LB medium with appropriate antibiotic and 0.1 mM IPTG to express the recombinant protein. Using low temperature induction (20 °C) for 24 h, the recombinant protein appeared mostly as the soluble protein (Fig. 2a). Through Ni-NTA purification procedures, EstGSU753 was eluted with the elution buffer (50 mM NaH_2_PO_4_, 300 mM NaCl, 100 mM imidazole, pH 8.0). The molecular weight of EstGSU753 observed in the electrophoregram determined to be approximately 30.0 kDa by SDS polyacrylamide gel electrophoresis (Fig. 2a) was consistent with that calculated (27.5 kDa).

### Biochemical characterization of the purified recombinant esterase

The ability of the purified enzyme to hydrolyze various substrates (*p*NP-ester C2–C16) was examined at 60 °C and pH 8.0. The substrate specificity towards *p*NP-esters of various fatty acids is shown in Fig. 2b. Compared with *p*NP-esters of longer-chain fatty acids, short-chain *p*NP-esters appeared to be preferred substrates. EstGSU753 showed the highest activity with *p*NP-caprylate (70 U/mg) among the esters examined. EstGSU753 showed no activity against *p*NP-laurate, *p*NP-myristate and *p*NP-palmitate.

The optimum temperature of the purified EstGSU753 was examined by assaying enzyme activity at different temperatures (30 °C, 40 °C, 50 °C, 60 °C, 70 °C and 80 °C) in 20 mM Tris-HCl buffer (pH 8.0). The purified EstGSU753 exhibited higher esterase activities over a temperature range of 50–70 °C (over 90% of the highest activity), among which the highest specific enzyme activity was at 60 °C **(**Fig. 2c**)**. The effect of pH on esterase activity was determined at various pH at 60 °C using *p*NP-caprylate as substrate. The purified EstGSU753 exhibited higher esterase activities over a pH range of 7.0–9.0, among which the highest specific enzyme activity was at pH 8.0. As shown in Fig. 2d, the activity of EstGSU753 decreased significantly below pH 6.0 and only about 20% of the maximal activity in this condition (pH 6.0).

### Stability in methanol at various concentrations

**Fig. 3:**
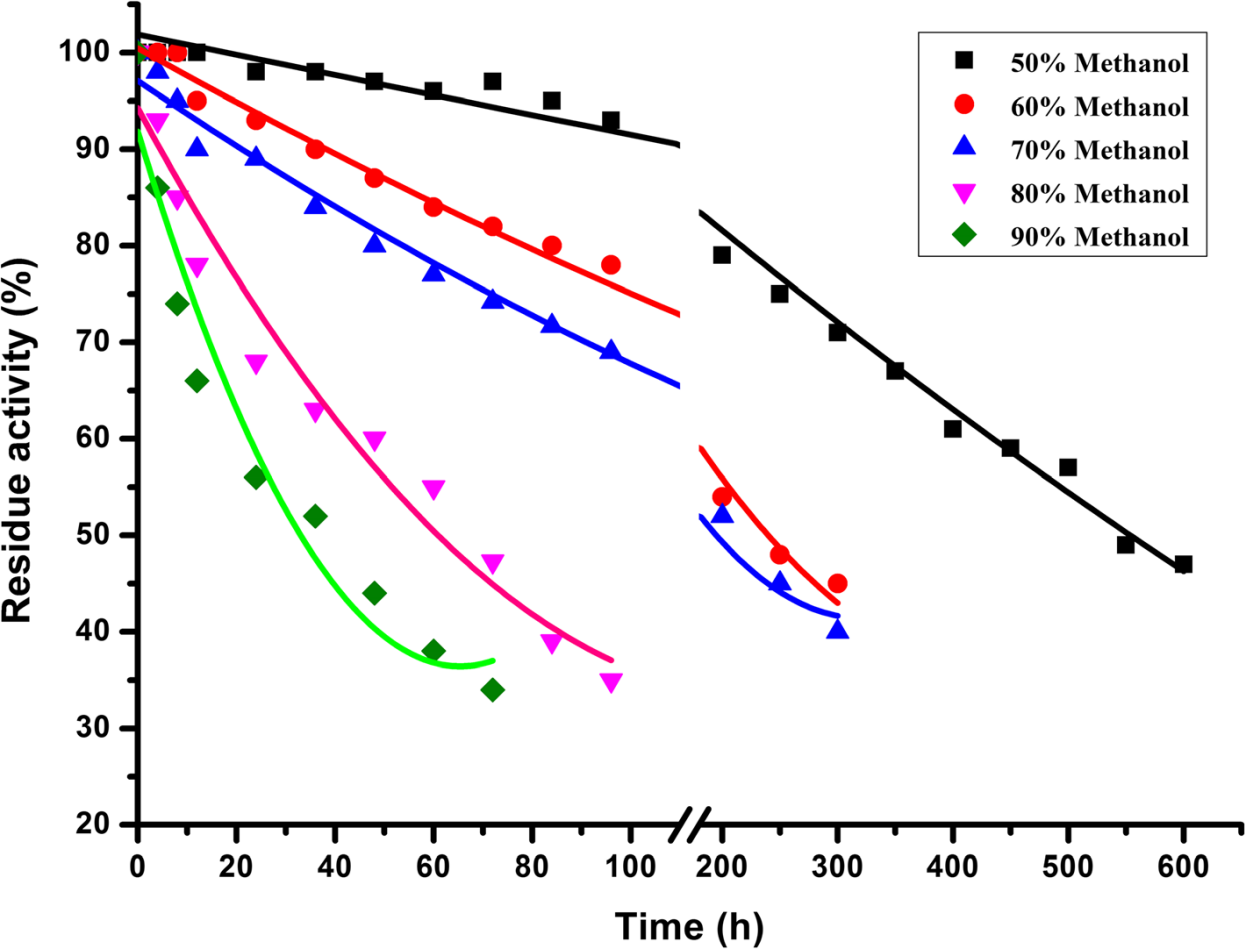
Methanol tolerance with different methanol concentrations

The stabilities of esterase EstGSU753 in high concentrations (50 to 90%) of hydrophilic solvents methanol, was evaluated by measuring their hydrolytic activities on *p*NP-caprylate after incubation intervals in methanol solutions (room temperature, 25 °C). The hydrolytic activity of the enzyme decreased as the solvent concentrations increased (Fig. 2). The half-life values of EstGSU753 were also determined from residual activity plots, which represent the deactivation profiles (Fig. 3). The t_1/2_ is the time required for the enzyme to lose half of its original activity under different conditions. Table 1 represents the t_1/2_ value calculated in different methanol concentration. The enzyme showed super-duper stability toward methanol, with t_1/2_ values of 33,300, 14,400, 11,640, 3650 and 1980 min for 50, 60, 70, 80 and 90% methanol, respectively. As shown in Fig. 3, EstGSU753 showed a steep decline in the residual activity as the methanol concentration increased. The most destructive effect was shown in 90% methanol, in which the residual activity dropped to less than 46% after 48 h of incubation (Fig. 3). However, EstGSU753 had elevated stability in 70% methanol, and maintained 46% of their activity after the maximal incubation time 600 h (Fig. 3).

**Table 1:** A comparison of the methanol-stable of EstGSu753 (this study) and *Geobacillus stearothermophilus* lipase T6/mutant at various methanol concentration

Items	t_1/2_ (min) (room temperature, 25 °C)				
	50% methanol	60% methanol	70% methanol	80% methanol	90% methanol
EstGSu753	33,300	14,400	11,640	3650	1980
lipase T6 [10]	347	173	4	–	–
T6: H86Y/A269T [10]	2429	1777	258	–	–

### Enzymatic transesterification reaction

**Fig. 4:**
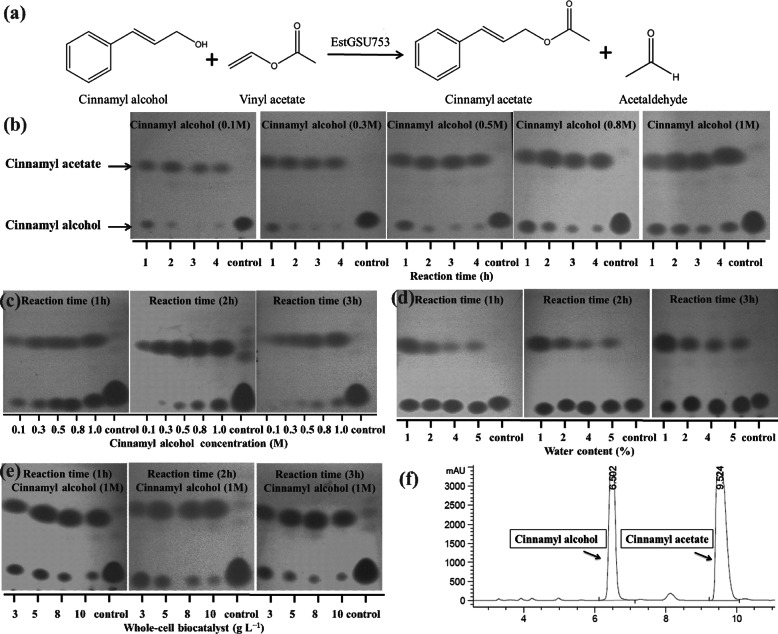
Cinnamyl acetate synthesis through transesterification in solvent-free system. (a) reaction equation of cinnamyl acetate synthesis. (b)(c) Effect of substrate concentration (0.1 M, 0.3 M, 0.5 M, 0.8 M, 1.0 M) and reaction time (lanes 1–4, reaction time 1–4 h) (TLC analysis). (d) Effect of addition of water (1, 2, 4, 5%) (TLC analysis). (e) Effect of addition of enzyme (TLC). (f) HPLC analysis of substrate and products

**Fig. 5:**
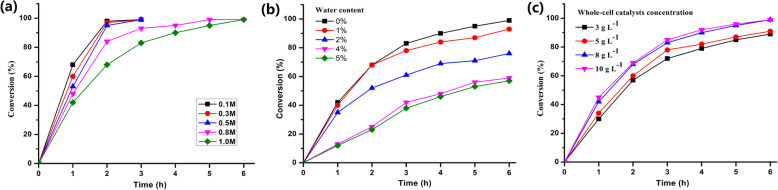
Quantitative analysis of cinnamyl acetate synthesis. (a) Effect of substrate concentration and reaction time. (b) Effect of addition of water (substrate: cinnamyl alcohol, 1.0 M). (c) Effect of addition of enzyme (substrate: cinnamyl alcohol, 1.0 M)

In this study, vinyl acetate was chosen as the acyl donor to synthesize cinnamyl acetate through transesterification (Fig. 4a) in a solvent-free system. The TLC analysis revealed that cinnamyl alcohol and cinnamyl acetate were detected in silica gel (Fig. 4bcde), and the results were further verified by HPLC analysis (Fig. 4f). As have a strong impact on the reaction course, and different substrate concentrations (0.1, 0.3, 0.5, 0.8 and 1.0 M) were investigated with different reaction time. The results were presented inFig. 4b, c. At the early stage, the conversion rates increased gradually at a relatively constant rate. Then the conversion rate slowed down and reached the maximum with the optimum reaction times varying from 2 (0.1 M) to 6 h (1.0 M). All the final conversion rates could reach to above 99% even if the substrate concentration was as high as 1.0 M. The effect of initial water was examined by adding water ranging from 1 to 10% (v/v) in the reaction mixture, and the cinnamyl alcohol (1.0 M) conversion was measured at interval time. Maximum molar conversion was obtained in absence of water, and it decreased with the increase in added water (Fig. 4d). On synthesis of cinnamyl acetate, the effect of enzyme amount was studied by varying from 3.0 g L^− 1^ to 10.0 g L^− 1^ (Fig. 4e). With the increase of enzyme amount (3 g L^− 1^ - 8 g L^− 1^), the reaction rate and conversion rate increased significantly. When the enzyme amount over more than 8 g L^− 1^, the reaction rate did not increase significantly, which may be due to the lack of substrate to access the active site of enzyme. HPLC analysis result shows that 99.2% molar conversion was achieved after 6 h of reaction at an enzyme concentration of 8.0 g L^− 1^ with 1.0 M substrate: cinnamyl alcohol (Fig. 5).

## Discussion

Esterase is an important enzyme and has been used especially in synthesis of new polymeric materials*.* Cloning the novel esterase gene from easily grown bacterium with distinct features is of interest for industrial applications [11]. *Geobacillus* species are well known for their ability to produce and secrete many useful extracellular enzymes in industrial application. Although many esterases and lipases from *Geobacillus* sp. have been intensively investigated, there was no report of esterase or lipase gene expression coding from *G. subterraneus*. In this work, the novel recombinant enzyme EstGSU753 with detailed enzymatic properties has preferable research significance. Homology analysis revealed that EstGSU753 in *G. subterraneus* DSM13552 shared the 60–80% identity with predicted triacylglycerol esterase in *Geobacillus* sp. The homology results reveal the remarkable research significance of the novel recombinant enzyme including enzyme characterizations. The substrate hydrolysis profile of the enzyme means that the recombinant esterase is an enzyme mainly active in short-chain fatty acid esters. Similar substrate specificity is found in a thermostable carboxylesterase from *Geobacillus* sp. ZH1. The catalytic efficiency was the greatest with pNPC4 among the *p*NP-esters with acyl chains of various lengths. These results indicate that EstGSU753 is not a lipase but an esterase. Esterases have preference for the short-chain length substrate, e.g., *G. thermoleovorans* ID-1 showed maximum activity of the enzyme toward p-NP caproate (C6), *G. thermocatenulatus* for butyrate and tributyrin (C4), *G. stearothermophilus* P1 for tricaprylin (C8) and caprate (C10) [12, 13]. The optimum temperature (60 °C) of the recombinant esterase is similar to that of esterase from *Geobacillus* sp. JM6 (60 °C) [14], *G. stearothermophilus* strain-5 (60–65 °C) [15] and *G. stearothermophilus* AH22 (50 °C) [16].

Organic solvent-stable esterases have great potential in commercial processes. The enzyme structure will be unstable in hydrophilic solvents, for the solvents may interact hinder hydrogen bonds and the active site pocket residues [17]. The resulting EstGSU753 showed remarkable stability against methanol. After the incubation at 50% methanol for 9 days, EstGSU753 retained 50% of its original activity. In recent years, few attempts were made to enhance lipases stability in methanol by applying protein engineering methods [18–20]. The reported literature generally determined the stability against methanol in low and medium methanol concentration in a certain range of 15–60%. Even incubation at 90% methanol for 2100 min, EstGSU753 retained 50% of its original activity. These results indicate that recombinant EstGSU753 was more methanol-stable than most of the reported esterases and lipases. A comparison of the methanol-stable of *G. stearothermophilus* lipase T6 and its mutant at various methanol concentration is shown in Table 1 [10].

Most of lipase-catalyzed reactions are carried out in organic solvents such as heptane, toluene or n-hexane [21, 22], and most of them are toxic and inflammable. The solvent-free system has many advantages such as low cost, easy downstream processing and has been developed to eliminate organic solvents problems. In a solvent-free system, Geng B et al. synthesized cinnamyl acetate using immobilized lipase Novozym 435 [23], Chang SW et al. synthesized hexyl laurate using Lipozyme IM-77 [24]. Tomke PD et al. focused on synthesis of cinnamyl acetate via transesterification in solvent free system [25]. In synthesizing various flavor esters, Bezbradica D et al. investigated the effect of substrate polarity on lipase activity in a solvent-free system [26].

## Conclusions

In this study, we obtained the novel esterase gene (*estGSU*753) from the strain *Geobacillus subterraneus* DSM13552 and the gene was functional expressed in *E. coli*. The esterase gene is the first report esterase gene cloning from *G. subterraneus.* Meanwhile, the detailed report of EstGSU753 enzymatic properties and the preliminary study of biocatalysis in cinnamyl acetate supplied the novel data for *G. subterraneus* esterase and lipase research. Also, the alkaline methanol-stable esterase reveals the potential value in industrial applications.

## Methods

### Strains, plasmids and chemicals

*Escherichia coli* DH5α and plasmid pMD19-T (TaKaRa, Shiga, Japan) were used for gene cloning and DNA sequencing. *Escherichia coli* BL21 and plasmid pET-28a (Novagen, Madison, WI, USA) were used to construct the protein expression strain. T4 DNA ligase, Ex-Taq DNA Polymerase, restriction enzymes and DNA/Protein marker were purchased from TaKaRa Bio. (Shiga, Japan). Kanamycin, chloromycetin, ampicillin and Isopropyl-β-D-thiogalactopyranoside (IPTG) were purchased from Amresco (Shanghai Genebase Co., Ltd., China). Plasmid Mini Prepare kit and DNA Mini kit were purchased from Axygen Biosciences (Union City, CA, USA). The substrates *p*NP-acetate (C2), *p*NP-butyrate (C4), *p*NP-hexanoate (C6), *p*NP-caprylate (C8), *p*NP-decanoate (C10), *p*NP-laurate (C12), *p*NP-myristate (C14) and *p*NP-palmitate (C16) were purchased from Fluka and Sigma (St. Louis, MO, USA). Other chemicals were obtained commercially and were of the reagent grade. *G. subterraneus* DSM13552 was obtained from Deutsche Sammlung von Mikroorganismen und Zellkulturen GmbH (DSMZ) and was grown aerobically at 60 °C in Luria Bertani (LB) medium. All the *E. coli* strains containing recombinant plasmids were cultured in LB at 37 °C.

### Cloning of the esterase gene and DNA manipulation

The *G. subterraneus* DSM13552 was harvested after overnight growth in LB medium and DNA extraction was performed using the DNA Mini kit. Based on the information of *Geobacillus* sp. esterase in GenBank, degenerate primers (GSU753-U: ATGAACVAACWATACHCTRTACTG. GSU753-D: TTATCCGSCGTGCYTGGHGAARAAT) for the esterase gene sequence were designed. The amplification protocol (initial denaturation at 95 °C for 2 min, 30 cycles of 95 °C for 1 min, 55 °C for 30 s, 72 °C for 1 min, and a final extension of 10 min at 72 °C) was used. The PCR products were cloned into pMD-l9T simple vector and then transferred into *E. coli* DH5α. Then the full-length esterase gene was obtained and sequenced. The primers (forward primer GSU753-EU (5′-GCGGGATCCATGAACGAACAATACCCTG-3′, reverse primer GSU753-ED (5′-CTAAAGCTTTTATCCGGCGTGCTTGGCG-3′, underlined nucleotides indicate restriction enzyme sites) were used for esterase gene amplification. After digestion by *Bam*H I/*Hin*d III, the PCR products were ligated into the pET-28a vector, which was digested by the same restriction endonuclease. Recombinant plasmids with the target gene were transformed into *E. coli* BL21 (DE3) cells and the recombinant strains were selected for further analysis.

### Heterogeneous expression/purification and SDS-PAGE analysis

The methods for enzyme expression and heterologous protein Ni-NTA purification procedures have been described previously. The recombinant esterase strain was incubated in 10 mL LB medium containing kanamycin (50 μg/mL) at 37 °C for 12 h–16 h and transferred into 100 mL LB medium for propagation. IPTG (0.1 mM) was added to the medium until the absorbance reached to 0.8 (OD 600 nm). After cultured at 20 °C for 24 h, the crude enzyme was collected by ultrasonic broken after the strains were harvested by centrifugation (12,000×g, 10 min). After lysed by sonication, the crude enzyme was passed through a 0.22 μm filter, and was applied to an equilibrated Ni-NTA column (1 mL, Qiagen) with the lysis buffer (NPI 10: 50 mM NaH_2_PO_4_, 300 mM NaCl, 10 mM imidazole, pH 8.0). Then the fusion protein (His-tagged EstGSU753) was eluted with a linear gradient of washing buffer (from NPI 50 to NPI 250). The purified protein was desalted and concentrated by ultrafiltration using a 25 mL Amicon Ultra Centrifugal Filter Device (10 kDa molecular weight cut-off, Millipore, USA). The crude extract and the purified enzyme were analyzed through SDS-PAGE. All purification steps were carried out at 4 °C or ice-water bath. Protein concentration of purified enzyme was determined by the Bradford method with bovine serum albumin (BSA) as the standard.

### Recombinant esterase enzymatic properties analysis

Enzyme activities of purified EstGSU753 solution were assayed by measuring the absorbance at 405 nm of p-nitrophenol. One unit (U) was defined as the amount of enzyme that liberated 1 μmol pNP per minute under the standard assay conditions. The reaction mixture (1.0 mL reaction system) contained 50 μL of 25 mM *p*NP-octoate solution, 900 μL buffer (50 mM Tris-HCl, pH 8.0) and 50 μL of appropriately diluted enzyme samples. The substrate specificity was detected using the following substrates *p*-nitrophenyl ester: *p*NP-acetate (C2), *p*NP-butyrate (C4), *p*NP-caproate (C6), *p*NP-octoate (C8), *p*NP-decenoate (C10), *p*NP-laurate (C12), *p*NP-myristate (C14) and *p*NP-palmitate (C16). The enzyme assay was carried out under standard assay conditions (pH 8.0, 60 °C). The optimum pH of EstGSU753 was evaluated over a range of pH 4 to 9 for 5 min at 60 °C. The following buffer systems were used in this study: pH 4.0–5.0, 100 mM citric acid-sodium citrate; pH 6.0–7.0, 100 mM sodium phosphate; pH 8.0, 50 mM Tris-HCl; pH 9.0, 50 mM glycine-NaOH. The optimum temperature of EstGSU753 was determined at different temperatures (30–80 °C) using *p*NP-octoate as substrate. The relative activity of EstGSU753 was used and the optimum conditions was considered as 100%.

### Half-life determinations in methanol

Half-life (*t*_1/2_) values of esterase EstGSU753 in 50, 60, 70, 80 and 90% methanol were calculated by measuring the residual activities after different incubation periods (room temperature, 25 °C). A 100 μL volume of esterase solution was mixed with methanol solution ranging from 50 to 90% to a final volume of 1 mL (for example, to make a 90% methanol solution, we combined 100 μL esterase solution and 900 μL methanol). The reaction was stopped periodically and the samples were detected by standard enzyme assay conditions. Each experiment was repeated three times.

### Cinnamyl acetate synthesis through transesterification in solvent-free system

To study the enzymatic properties of esterase EstGSU753, cinnamyl acetate was synthesized through the transesterification of cinnamyl alcohol with vinyl acetate as the acyl donor. The whole-cell catalyst was obtained by strain precipitation centrifugation and freeze-drying. In the experiment, the single factor experiment was used to optimize the reaction conditions including substrate cinnamyl alcohol concentration (0.1 M, 0.3 M, 0.5 M, 0.8 M, 1.0 M, the mole ratios between vinyl acetate and cinnamyl alcohol is 3:1), reaction time (1–6 h), water content (1, 2, 4, 5% (v/v)) and whole-cell catalyst (3.0 g L^− 1^ to 10.0 g L^− 1^). The reaction was carried out under optimal reaction conditions and without use of any other additional solvent. With the ultrasound assisted and mechanically agitated, the enzymatic transesterification reaction was carried out using 50 cm^3^ flat bottom glass reactor, which was immersed in an ultrasonic thermostatic water bath. Using different concentration of the substrate cinnamyl alcohol and 8 g L^− 1^ whole-cell catalyst, standard transesterification assays were detected in a final volume of 15 mL completed with vinyl acetate (60 °C, 200 rpm). 20 μL reaction samples were diluted 20 times in vinyl acetate for further analysis. A parallel reaction without the enzyme was used as the control. The conversion rate (%) was detected from the conversion of alcohol to ester.

### The products analysis

The transesterification products were analyzed using thin-layer chromatography (TLC) and HPLC. Silica gel plates were used for TLC analysis. After activated at 110 °C for 30 min, product samples were spotted onto the TLC plate. The plate was placed in a TLC chamber using n-hexane and ethyl acetate (80:20) as the solvent system. After the TLC plate was dried, the products bands were visible at 254 nm. For HPLC analysis, samples were loaded on an OB-H capillary column using 90% n-hexane and 10% isopropyl alcohol (v/v) as the mobile phase and a flow rate of 1 mL/min at 25 °C. Ester samples were detected at 254 nm and identified by comparing retention times with standard analysis method. Through dividing the slope of standard curves and measuring area under the curve for each identified compound, quantification of every materials was performed.

### Sequence accession number

The sequence of *G. subterraneus* DSM13552 esterase gene *estGSU*753 is available in the GenBank database under the accession number KY426947.

## Data Availability

The datasets used and/or analyzed during the current study are available from the corresponding author on reasonable request.

## Abbreviations

*estGSU*753: the esterase gene from *Geobacillus subterraneus* DSM13552
EstGSU753: the esterase from *Geobacillus subterraneus* DSM13552;
LB: Luria Bertani medium
IPTG: Isopropyl-β-D-thiogalactopyranoside;
SDS-PAGE: Sodium dodecyl sulfate polyacrylamide gel electrophoresis
TLC: Thin layer chromatography
HPLC: High performance liquid chromatography.
